# Nurse-patient interaction in hospital care: a cross-sectional study in Peruvian nurses

**DOI:** 10.15649/cuidarte.4971

**Published:** 2026-03-25

**Authors:** Katherine Jenny Ortiz-Romaní, Miriam Lizbeth Solis-Mallqui, Yonathan Josué Ortiz-Montalvo

**Affiliations:** 1 Universidad Católica Sedes Sapientiae, Lima, Perú. E-mail: kathyortiz95@gmail.com Universidad Católica Sedes Sapientiae Lima Perú kathyortiz95@gmail.com; 2 Universidad Católica Sedes Sapientiae, Lima, Perú. E-mail: 2019100926@ucss.pe Universidad Católica Sedes Sapientiae Lima Perú 2019100926@ucss.pe; 3 Universidad Privada del Norte, Lima, Perú. E-mail: Universidad Privada del Norte Lima Perú yonatanortiz79@gmail.com

**Keywords:** Interpersonal Relations, Nursing Care, Hospitalization, Cross-Sectional Studies, Relaciones Interpersonales, Cuidados de Enfermería, Hospitalización, Estudios Transversales, Relações Interpessoais, Cuidados de Enfermagem, Hospitalização, Estudos Transversais

## Abstract

**Introduction:**

Nurse-patient interaction is key to humanized care and improved health outcomes; therefore, updated quantitative studies in hospitals are needed.

**Objective:**

To evaluate the levels of nurse-patient interaction and associated factors among nursing professionals.

**Materials and Methods:**

A cross-sectional analytical study was conducted with 70 nurses from a public hospital in Lima, Peru. The Caring Nurse-Patient Interactions (CNPI-70) was used, which comprises ten dimensions: humanism, hope, sensitivity, helping relationship, expression of emotions, problem-solving, teaching, environment, needs, and spirituality. The instrument, originally developedby Cossetteetal. hasreported Cronbach’salphacoefficients ranging from 0.73 to 0.91. Personal factors were also considered, and bivariate and multivariate analyses were performed.

**Results:**

48.57% of nurses demonstrated a moderate level of caring interaction with their patients, indicating a basic functional relationship but lacking emotional depth. Factors associated with caring interactions were having a family member with a chronic illness (aPR = 1.72; 95% CI: 1.01-2.94) and the number of jobs (aPR = 1.84; 95% CI: 1.08-3.12).

**Discussion:**

The level of nurse-patient interaction in this study is similar to that reported by other studies in public hospitals, yet differs from that of private hospitals. Regarding the number of jobs, this may be because nurses acquire more experience, develop interpersonal skills, and develop confidence.

**Conclusion:**

The predominant level of nurse-patient interaction was moderate. The factors associated with greater interaction were having a family member with a chronic illness and the number of jobs held.

## Resumen

**Introducción:**

La interacción enfermera-paciente es clave para un cuidado humanizado y mejores resultados en salud, por lo que se requieren estudios cuantitativos actuales en hospitales.

**Objetivo:**

Evaluar los niveles de interacción enfermera-paciente y los factores asociados en profesionales de enfermería.

**Materiales y métodos:**

En el estudio transversal analítico se consideró a 70 enfermeros de un hospital público de Lima-Perú. Se utilizó la escala Caring Nurse-Patient Interactions (CNPI-70) la cual cuenta con diez dimensiones: humanismo, esperanza, sensibilidad, relación de ayuda, expresión de emociones, resolución de problemas, enseñanza, ambiente, necesidades y espiritualidad. Fue creada originalmente por Cossette et al., con un alfa de Cronbach entre 0,73 y 0,91. Asimismo, se consideró a los factores personales y se empleó un análisis bivariado y multivariado.

**Resultados:**

El 48,57% de los enfermeros tuvo un nivel moderado de interacción con sus pacientes, es decir existe una relación funcional básica, pero sin profundidad emocional. Además, los factores asociados a la interacción fueron tener un familiar con una enfermedad crónica (RPa:1,72; IC 95%: 1,01-2,94) y cantidad de empleos (RPa:1,84; IC 95%: 1,08-3,12).

**Discusión:**

El nivel de interacción enfermera-pacientes del estudio es similar al de otros estudios realizados en hospitales públicos, pero a la vez diferente al de los hospitales privados. Respecto a la cantidad de empleos, posiblemente es porque los enfermeros adquieren mayor experiencia, desarrollan habilidades interpersonales y seguridad.

**Conclusiones:**

Se evidencia que el nivel predominante de interacción enfermera-paciente es moderado, y los factores asociados son tener un familiar con una enfermedad crónica y la cantidad de empleos.

## Resumo

**Introdução:**

A interação enfermeiro-paciente é fundamental para um cuidado humano e melhores resultados de saúde, sendo necessários estudos quantitativos atuais em hospitais.

**Objetivo:**

Avaliar os níveis de interação enfermeiro-paciente e os fatores associados entre profissionais de enfermagem.

**Materiais e métodos:**

Este estudo transversal e analítico incluiu 70 enfermeiros de um hospital público em Lima, Peru. Foi utilizada a Escala de Interações Enfermeiro-Paciente no Cuidado, classificada em 10 dimensões: humanismo, esperança, sensibilidade, relação de ajuda, expressão de emoções, resolução de problemas, ensino, ambiente, necessidades e espiritualidade. Foi originalmente desenvolvida por Cossette et al., com um alfa de Cronbach entre 0,73 e 0,91. Fatores pessoais também foram considerados, e análises bivariadas e multivariadas foram realizadas.

**Resultados:**

48,57% dos enfermeiros apresentaram um nível moderado de interação com seus pacientes, o que significa que mantinham um relacionamento funcional básico, mas careciam de profundidade emocional. Além disso, os fatores associados à interação foram ter um familiar com doença crônica (aPR: 1,72; IC 95%: 1,01-2,94) e número de empregos (aPR: 1,84; IC 95%: 1,08-3,12).

**Discussão:**

O nível de interação enfermeiro-paciente neste estudo é semelhante ao de outros estudos realizados em hospitais públicos, mas também diferente em hospitais privados. Em relação ao número de empregos, isso pode ser porque os enfermeiros adquirem mais experiência, desenvolvem habilidades interpessoais e desenvolvem confiança.

**Conclusões:**

É evidente que o nível predominante de interação enfermeiro- paciente é moderado, e os fatores associados são ter um familiar com doença crônica e o número de empregos.

## Introduction

Nurse-patient interaction is more than an exchange of information: it is a key aspect of holistic care that includes physical and psychosocial dimensions^1^, as outlined in Jean Watson’s Theory of Human Caring from the perspective of the ten Carative factors or Caritas processes. This theorist promotes these processes as a guide for care practices, enabling nursing staff to plan, deliver, and evaluate care that addresses patients’ individual needs, including their spiritual needs^2^,^3^.

From Jean Watson’s perspective, nurse-patient interaction becomes an experience of deep connection. This relationship, based on empathy, presence, and respect, helps patients feel valued and as unique human beings. It also alleviates their suffering, improves their well-being, and strengthens their confidence in the care process. Moreover, nurse-patient interaction benefits nurses themselves because this type of interaction provides an opportunity for personal and professional growth. By caring with love, compassion, and mindfulness, nurses find greater meaning and purpose in their work, reinforcing their sense of vocation and job satisfaction^4^-^6^. However, some authors have mentioned that hospitalized patients had had negative experiences and were dissatisfied with nursing professionals’ care^7^. They even report that therapeutic communication with nurses is limited^8^, often due to nursing staff shortages, heavy workloads, and poor hospital environments^9^,^10^, especially in rural hospitals^11^.

Several authors mention that, in order to understand nurse-patient interaction, it is important to assess nursing staff directly, as their perceptions may differ from those of patients^5^,^12^,^13^. Although it is difficult to measure nurse-patient interaction quantitatively from the standpoint of humanized care behaviors, some specific measurement instruments based on nursing theory have been developed, which can serve as self-assessment tools for nurses^5^,^14^. Only some studies show that nurses working in hospital settings exhibit low or moderate levels of interaction^12^,^13^. Among the care dimensions least frequently demonstrated by nurses during interactions with patients is spirituality^13^-^16^, as some nurses focus only on physical needs while avoiding conversations about the patient’s suffering and not acknowledging their beliefs. Another dimension that is least frequently demonstrated is the expression of both positive and negative feelings, as nurses may interrupt or ignore patients’emotional expressions (e.g., crying or joy), respond with scripted phrases, or minimize patients’ feelings, which results in a cold and distant relationship^17^.

A deeper understanding of this phenomenon is important. To this end, further studies are needed on nursing staff to explore both personal and work-related factors, as well as health and family history, as these aspects can negatively affect nurses’ physical and emotional health, limiting their ability to establish empathetic, close, and caring relationships^18^,^19^. Currently, there is scarce scientific evidence from public hospitals in Peru, particularly in Lima, where nurse-patient interaction occurs in settings characterized by high patient demand and heavy workloads due to limited human and material resources^20^. Such environments make it difficult for nursing staff to establish empathetic, communicative, and person-centered relationships, which affects the quality of care. In particular, they affect the dimensions or ten Carative factors proposed in Jean Watson’s Theory of Human Caring. In addition, these public hospitals serve vulnerable populations: patients with chronic diseases, low income, limited education, and, in many cases, from rural or peripheral areas^7^,^21^. Therefore, the objective of this study was to evaluate nurse-patient interaction and associated factors among nursing professionals working in a public hospital in Peru.

## Materials and Methods

**Study design and population**

A cross-sectional observational study with a quantitative and correlational design was conducted at Sergio E. Bernales Hospital in 2024. This public hospital is classified as level III-1 and provides healthcare services to approximately one million inhabitants across five districts and one province of Lima. It includes inpatient wards, critical care units, outpatient clinics, a sterile processing department, and a surgical center^20^. The study considered conducting a population census of the 116 nursing professionals with care responsibilities; however, due to the established inclusion and exclusion criteria, the effective sample size was 70. The study included male and female nurses who performed direct care activities in hospitalization, surgery, gynecology, internal medicine, pulmonology, trauma, and mental health units. Nursing professionals working in neonatology, pediatric surgery, or pediatrics were excluded, as the questionnaire items inquire about direct verbal communication with patients. Nurses who declined participation or failed to complete the questionnaire were also excluded. Therefore, a census sampling method was used, yielding a total of 70 participants.

**Measurement of nurse-patient interaction**

The main variable was caring nurse-patient interaction, comprising ten dimensions: humanism, hope, sensitivity, helping relationship, expression of emotions, problem-solving, teaching, environment, basic needs, and spirituality. This classification of ten dimensions was based on Jean Watson’s ten Carative factors^2^,^3^. The overall interaction score was categorized as low, moderate, and high, based on percentile distribution. Data were collected using the Caring Nurse-Patient Interactions (CNPI-70) scale, originally developed by Cosette et al.^22^ in a study involving 13 expert nurses. The scale consists of 70 items grouped into ten Carative factors or Caritas processes. Reported Cronbach’s alpha coefficients ranged from 0.73 to 0.91 across subscales, with inter-subscale correlations between 0.53 and 0.89. Pearson’s correlation coefficients between the subscales and social desirability ranged from -0.02 to 0.32, suggesting low to moderate bias. Likewise, subsequent versions of the scale were validated in terms of face and content validity in Spanish-speaking contexts^23^,^24^. Finally, the instrument was adapted to the Peruvian context, resulting in a 51-item version using a five-point Likert scale (1 = Not at all, 2 = A little, 3 = Moderately, 4 = A lot, 5 = Extremely).

This version of the instrument was developed ad hoc from the original^22^ and Spanish adaptations^23^,^24^, incorporating linguistic and cultural modifications for the Peruvian context. Five expert judges in the field reviewed the instrument to assess content validity and confirm language clarity and comprehensibility. Although this validation has not been published separately, it was essential for adapting the tool. No pilot testing was conducted; however, internal consistency of the instrument was evaluated in the total sample, yielding a Cronbach’s alpha of 0.97, which indicates high internal consistency. It should be noted that the CNPI-70 is a highly consistent and applicable instrument for clinical nurses, allowing measurement of their perceptions regarding the importance, competence, and applicability of caring nurse-patient interaction^13^.

**Measurement of covariates**

The personal factors of nursing professionals included age (continuous variable); sex (male and female); presence of a family member with a chronic illness (yes, no); presence of health problems (yes, no); years of work experience in the current service (continuous variable); teaching status (yes, no); number of healthcare-related jobs (one, two); highest educational level attained (bachelor’s degree, second specialty, and master’s degree); participation in hospital-based training in humanized care within the past six months (yes, no); receipt of verbal recognition from the head nurse or service supervisor (yes, no); and number of patients cared for per day (continuous variable).

**Data analysis**

A database was created in Microsoft Excel, and analyses were performed using Stata version 17. Results were summarized in statistical tables. For categorical variables, frequencies and percentages were calculated; for continuous variables, means and standard deviations were reported. In the bivariate analysis, the Chi-square test was applied to categorical variables. The Shapiro-Wilk test was used to assess normality, and later select the Kruskal-Wallis test. To avoid overestimation of associations, Poisson regression was performed to calculate prevalence ratios (PRs). An adjusted multivariate model was subsequently constructed, excluding variables that demonstrated multicollinearity or a Hosmer–Lemeshow goodness-of-fit test value below 0.05. In statistical analysis, a p-value ≤ 0.05 was considered statistically significant. The complete dataset supporting this analysis is available in Mendeley Data^25^.

**Ethical considerations**

The study was approved by the Institutional Research Ethics Committee of the participating hospital (Approval No. 0117). In addition, signed informed consent was requested, and anonymity of participants was maintained, as was the data securely stored on a private computer.

## Results

Among the main descriptive findings, the mean age of nursing professionals was 42 years. In total, 38.57% reported having a family member with a chronic illness, 32.86% held two jobs, 85.71% had not received any training at the hospital, and 70.00% had not received verbal recognition from their supervisor. The average number of patients cared for per day was 20. Additional variables are presented in Table 1.

**Table 1:** Description of nurses’ personal factors. n=70

Personal factors	% (n)
Age. Mean ± SD	42.11 ± 9.68
Sex	
Male	14.29 (10)
Female	85.71 (60)
Having a family member with a chronic disease	
No	61.43 (43)
Yes	38.57 (27)
Having health problems	
No	94.20 (65)
Yes	5.80 (4)
Years working in the current service Mean ± SD	8.46 ± 6.79
Teacher	
No	75.71 (53)
Yes	24.29 (17)
Number of healthcare jobs	
One	67.14 (47)
Two	32.86 (23)
Academic degree attained	
Bachelor’s degree	20.00 (14)
Second specialty	67.14 (47)
Master’s degree	12.86 (9)
Received training at the hospital in the last six months.	
No	85.71 (60)
Yes	14.29 (10)
Received verbal recognition from supervisor	
No	70.00 (49)
Yes	30.00 (21)
Number of patients cared for per day Mean ± SD	20.75 ± 7.30

SD: Standard deviation

As shown in Table 2, nearly half of the nursing professionals (48.57%) demonstrated a moderate level of nurse-patient interaction, indicating a relationship that was functional but lacked emotional depth.

**Table 2:** Description of the level of nurse-patient interaction. n=70

Level	% (n)
Low	25.71 (18)
Moderate	48.57 (34)
High	25.71 (18)

Regarding the importance attributed to the ten Caritas processes, Table 3 shows that the lowest mean scores were observed in the processes of basic needs (mean = 17.97; range:13-25 points) and spirituality (mean = 18.46; range:15-25 points).

**Table 3:** Description of the ten Caritas Processes

Process	Mean ± SD	Min-Max
Humanism	20.91 ± 2.47	15-25
Hope	18.61 ± 3.08	14-25
Sensitivity	18.86 ± 3.03	13-25
Helping relationship	21.4 ± 3.13	16-28
Expression of emotions	17.73 ± 2.81	13-25
Problem solving	18.91 ± 3.09	13-25
Teaching	18.34 ± 2.93	12-25
Environment	17.3 ± 3.03	12-25
Basic needs	17.97 ± 3.07	13-25
Spirituality	18.46 ± 2.83	15-25

SD: Standard deviation, Min: Minimum, Max: Maximum.

A bivariate analysis between personal factors and nurse-patient interaction revealed statistically significant associations for having a family member with a chronic illness, being a teacher, and the number of jobs. These results were observed in the crude analysis using prevalence ratios (PRs). The remaining results are presented in Table 4.

**Table 4:** Bivariate analysis between personal factors and nurse-patient interaction

Personal factors	Level of nurse-patient interaction			p-value	CPR (95% CI)	p-value
	Low % (n)	Moderate % (n)	High % (n)			
Age*	44.28 ± 11.78	41.85 ± 9.46	40.44 ± 7.74	0.732	0.99 (0.96 - 1.01)	0.392
Sex				0.889		
Male	30.00 (3)	50.00 (5)	20.00 (2)		1	-
Female	25.00	48.33 (29)	26.67 (16)		1.13 (0.56 - 2.27)	0.733
Having a family member with a chronic disease				<0.001		
No	39.53 (17)	51.16 (22)	9.30 (4)		1	-
Yes	3.70 (1)	44.44 (12)	51.85 (14)		2.12 (1.32 - 3.41)	0.002
Having health problems						0.066
No	26.15 (17)	50.77 (33)	23.08 (15)		1	-
Yes	0.00 (0)	25.00 (1)	75.00 (3)		1.81 (0.83 - 3.94)	0.138
Years working in the current service *	9.66 ± 7.20	7.85 ± 6.79	8.38 ± 6.58	0.647	0.99 (0.96 - 1.03)	0.683
Teacher				<0.001		
No	30.19 (16)	58.49 (31)	11.32 (6)		1	-
Yes	11.76 (2)	17.65 (3)	70.59 (12)		1.96 (1.21 - 3.17)	0.006
Number of healthcare jobs				<0.001		
One	36.17 (17)	55.32 (26)	8.51 (4)		1	-
Two	4.35 (1)	34.78 (8)	60.87 (14)		2.16 (1.35 - 3.46)	0.001
Academic level attained				0.041		
Bachelor’s degree	21.43 (3)	71.43 (10)	7.14 (1)			-
Second specialty	25.53 (12)	48.94 (23)	25.53 (12)		1.17 (0.62 - 2.20)	0.634
Master’s degree	33.33 (3)	11.11 (1)	55.56 (5)		1.43 (0.63 - 3.23)	0.395
Received training at the hospital in the last six months				0.468		
No	28.33 (17)	46.67 (28)	25.00 (15)		1	-
Yes	10.00 (1)	60.00 (6)	30.00 (3)		1.24 (0.67 - 2.31)	0.495
Received verbal recognition from supervisor				0.631		
No	22.45 (11)	51.02 (25)	26.53 (13)		1	-
Yes	33.33 (7)	42.86 (9)	23.81 (5)		0.87 (0.51 - 1.47)	0.602
Patients attended per day *	21.33 ± 4.35	21.82 ± 9.04	18.17 ± 5.37	0.179	0.98 (0.95 - 1.02)	0.347

CPR: Crude prevalence ratio, CI: Confidence interval. * Kruskal-Wallis test, values expressed as mean ± standard deviation.

According to the adjusted analysis shown in Table 5, nursing professionals who reported having a family member with a chronic illness were 72% more likely to exhibit greater interaction with hospitalized patients compared with those who did not (aPR = 1.72; 95% CI: 1.01–2.94; p=0.048). Similarly, greater nurse-patient interaction was observed in nurses with two jobs compared with those with only one job (aPR 1.84, 95% CI 1.08-3.12, p-value: 0.024). The model was adjusted for age, sex, years of service, training in humanized care, and verbal recognition.

**Table 5:** Multivariate analysis of personal factors and nurse-patient interaction

Personal factors	Level of nurse-patient interaction	
	aPR (95% CI)	p-value
Family member with a chronic disease *		
No	1	-
Yes	1.72 (1.01 - 2.94)	0.048
Number of healthcare jobs*		
One	1	-
Two	1.84 (1.08 - 3.12)	0.024

aPR: adjusted prevalence ratio, CI: Confidence Interval. * Adjusted for age, sex, years of service, training in humanized care, and verbal recognition

## Discussion

In this study conducted in 2024, a large proportion of nursing professionals demonstrated a moderate level of interaction with their patients. This result bears some similarity to findings from research conducted in Jordan during the COVID-19 pandemic, in which hospital nurses also demonstrated moderate interaction levels. In that context, influencing factors included family discouragement of direct contact, working conditions, high patient-to-nurse ratios, available biosecurity resources, and insufficient knowledge about the virus^12^. Conversely, studies conducted in two private hospitals in Istanbul, Turkey^26^, and Croatia^13^ reported good interaction. One plausible explanation for these discrepancies in results is that private hospitals have adequate staffing and institutional policies that promote high-quality patient care^27^. In contrast, the present study was conducted in a public hospital, where many nurses care for about 20 patients per day and receive neither recognition from their supervisors for their hard work nor institutional training in humanized care.

Regarding nurse-patient interaction measured through Jean Watson’s ten Caritas processes, the lowest mean scores were observed in the dimensions related to the satisfaction of basic human needs and spirituality. This finding contrasts with other research in which patients’ basic needs are considered a priority within nurse-patient interaction^13^,^15^. However, regarding spirituality, the results are consistent with other studies reporting low ratings for this dimension among nursing staff^13^,^15^,^16^. From Watson’s perspective, responding to or attending to these Caritas processes is essential to the expression of humanized care. The low scores in these areas suggest a disconnect between theoretical ideals and everyday practice, possible due to heavy workloads and reductionist conceptions of care among some professionals. While Watson advocates for holistic nursing centered on the human being as a whole, in practice, some nurses restrict their role to technical or purely physical aspects. Consequently, one of the enduring challenges for nursing practice is how to effectively integrate the Theory of Human Caring into healthcare contexts characterized by heavy care demands and structural limitations^28^.

In relation to associated factors, the multivariate analysis revealed that having a family member with a chronic illness was associated with a higher likelihood of demonstrating better nurse-patient interaction. Currently, there is no quantitative evidence to support this finding. However, there is a possible explanation for this result, as close contact with the needs and suffering of a chronically ill relative exposes nurses to the physical, emotional, and social impact of illness^29^,^30^. Such lived experience fosters greater sensitivity, compassion, and empathy, enabling nurses to provide more understanding and emotionally attuned care. Moreover, nurses with this experience may strengthen their professional commitment and understand the importance of interacting with their patients through better communication and dignified treatment^31^.

Another important finding was that nurses working twojobs were more likely to display moderate levels of interaction with patients. Although no studies were found that directly examine this association, one possible explanation relates to the breadth of experience gained in diverse care environments. From the perspective of Jean Watson’s Theory of Human Caring, the professional’s ability to establish deep human relationships through acts of love and compassion is underscored. Working in more than one institution may expose nurses to a broader range of clinical and human situations, thereby enriching their communication and empathy skills. This interpretation aligns with findings from a study conducted in a hospital in Vietnam, where longer employment duration was associated with improved nurse-patient communication, attributed to the accumulation of experience^32^. In this sense, it may not be the number of jobs per se that determines better interaction, but rather the opportunity to develop interpersonal skills through exposure to diverse clinical settings. However, further research is warranted to explore this relationship in greater depth^33^.

This study had some limitations. First, a self-report instrument was used to assess nurse-patient interaction, which may have introduced social desirability bias or subjective perceptions on the part of participants. These biases could have affected the accuracy with which actual interactions were represented. Future research should consider employing direct observation methods in clinical settings, either through structured techniques (e.g., checklists) or unstructured approaches (e.g., participant observation) to obtain a more comprehensive and objective view of the phenomenon. Second, there is little evidence of studies applying this instrument and examining its association with nurses’ personal factors within the Peruvian context, which restricted comparison with previous research. Finally, the population was small. Due to heavy workloads, many nurses made an effort to complete the extensive questionnaire, and some eventually withdrew from the study.

## Conclusions

This study found that the predominant level of caring nurse-patient interaction is moderate. Regarding nurses’ personal factors, having a family member with a chronic illness and holding two jobs were associated with a greater likelihood of engaging in more caring interactions with hospitalized patients. In addition, the research yielded relevant findings that highlight the need to strengthen nurse-patient interaction, particularly in the dimensions of spirituality and attention to basic human needs, which obtained the lowest scores.

These results have important practical implications for hospital settings. First, it is recommended that institutions adopt a holistic approach to care that views the patient as an integrated being (body, mind, and spirit) in accordance with Jean Watson’s Theory of Human Caring. Continuing education programs for nursing professionals should therefore incorporate specific content on spirituality, empathy, and the identification and satisfaction of basic needs, even in high-demand clinical settings. Moreover, institutional policymakers should develop strategies to enhance nurses’ self-efficacy by providing regular training in humanized care, as well as by implementing work incentives, organizational support, and conditions that allow sufficient time and space to build meaningful relationships with patients.
